# It’s Time to Run!

**DOI:** 10.3390/jcm12175758

**Published:** 2023-09-04

**Authors:** Gaia Cattadori, Anna Picozzi, Silvia Di Marco

**Affiliations:** IRCCS Multimedica, 20138 Milan, Italy; anna.picozzi@multimedica.it (A.P.); silvia.dimarco@multimedica.it (S.D.M.)

## 1. Introduction

Several epidemiological studies have consistently reported inverse associations between cardiorespiratory fitness and the risks of cardiovascular disease and mortality [1,2], leading to an improvement of physical activity in the general population and to a growing interest in a scientific approach to this topic. Moreover, representing a profound change to the historical prescription of rest in cardiac patients, exercise training is now considered be the main therapy [3,4,5].

Consequently, cardiac rehabilitation (CR), with exercise training as a core component, is widely proposed for primary and secondary cardiovascular prevention [6,7]. CR refers to a complex and multidisciplinary intervention (exercise training, counselling for exercise/health/nutrition/cardiovascular risk factors and psychological support) personalized to patients with heart disease [8]. Despite strong guidelines and recommendations, global access to CR is persistently poor [8]. In “Rehabilitation 2030: a call for action”, the WHO’s Department of Noncommunicable Diseases builds an evidence-based Package of Rehabilitation Interventions, in combination with suggestions for supplying their delivery. Module 4 addresses “Cardiopulmonary condition” [9].

Cardiopulmonary exercise testing permits the simultaneous evaluation of the ability of the cardiovascular and respiratory systems to perform gas exchange in order to support the increase in muscle respiration required to perform exercise. Knowing the physiology or even the pathophysiology of exercise performance and/or limitations is crucial for safe cardiac rehabilitation, to establish exercise training protocols and to assess the final response [10,11].

Based on the above, the aim of this Special Issue is to explore new insights into cardiopulmonary exercise testing and cardiac rehabilitation, provided below in three brief topic chapters summarising the main ”take home messages”.

## 2. Indications

Kleczynski and colleagues [12] measured clinical performance and Quality of Life modifications in patients treated with **transcatheter aortic valve replacement (TAVR)** who were transferred to an inpatient cardiac rehabilitation (CR group) or discharged home (DH group) to evaluate the impact of CR in TAVR patients during a 1-year follow-up period. Data are scarce in this field. The CR consisted of exercise prescriptions, psychosocial interventions and lifestyle modifications, overseen by a multidisciplinary team (physicians, nurses, physiotherapists, dieticians and social workers, if necessary). Data show that CR led to significant improvements in clinical performance and Quality of life in the TAVR patients, offering the opportunity to provide specific recommendations. Moreover, the idea to periodically offer CR to TAVR patients is suggested by evidence that the outcomes were attenuated after 1 year of follow-up.

It is now widely demonstrated that SARS-CoV2 infection does not end with control over pulmonary inflammation, as indicated by a negative virology test. Rehabilitation centres could be now useful for **COVID-19 patients**, mainly due to the positive effects of exercise training on not only the immune system and inflammation but also on the multifactorial features characterizing post-COVID-19 patients’ clinical conditions, including pulmonary, cardiac, vascular and musculoskeletal dysfunction.

We therefore reviewed [13] previous papers addressing exercise training and consequently conceived a proposal for post-COVID-19 patients, joining the recommended exertional programs for:

- COVID-19 patients (very little data);
- Severe acute respiratory syndrome (SARS) patients (who likely have similar damage to lungs and other organs as COVID-19 patients);
- Frail patients;
- Patients with interstitial lung disease and, in particular, idiopathic pulmonary fibrosis;
- Patients with chronic obstructive pulmonary disease (if applicable, based on a detailed preliminary evaluation);
- Patients with pulmonary hypertension (if applicable, based on a detailed preliminary evaluation);
- Patients with heart failure (if applicable, based on a detailed preliminary evaluation).

Therefore, the “new combined COVID-19 exercise protocol” was tailored to post-COVID-19 patients who are imagined as frail subjects with interstitial lung disease complicated by a mixture of pulmonary, cardiac and vascular diseases [13].

## 3. Protocols

**Resistance exercise (RE)** remains underused, with poor specific guidelines and persistent safety doubts about the enhancement of cardiovascular risk. In this Special Issue, Kambic and colleagues [14] published the first study on the safety, the feasibility and the efficacy of high-load RE (HL-RE) and low-load RE (LL-RE) combined with aerobic exercise and compared with AE alone in the early phase of CR in patients with coronary artery disease. The data showed the safety of RE (with haemodynamic responses within the physiological range) and the improvement in exercise tolerance for both HL-RE and LL-RE. LL-RE was better tolerated than HL-RE, while a greater maximal muscle strength improvement was seen in HL-RE than in LL-RE.

Training zone classification is a cornerstone for the assessment of functional capacity for CR exercise protocols, and the identification of the **aerobic threshold**, usually measured via cardiopulmonary exercise testing, is crucial [11]. In patients with heart failure and ischemic vascular disease, Rogers and colleagues [15] demonstrated a close relationship between the heart rate variability threshold based on fractal correlation properties (DFA a1) with the first ventilator threshold. Moreover, the data evidenced similar changes in the two thresholds after an exercise training program, suggesting the use of heart rate variability for identifying the anaerobic threshold in the cardiac disease population.

Physical exercise involves the interaction of the pulmonary, cardiovascular and muscle systems. Peak oxygen consumption, the gold standard for performance evaluation, is measured via cardiopulmonary exercise testing as ventilation multiplied by the inspired oxygen concentration minus the expired oxygen concentration, reflecting the total oxygen extraction relating to cardiopulmonary–muscle crosstalk in the body. Moreover, peak oxygen consumption, following the Fick principle, corresponds to the product of cardiac output and the arteriovenous oxygen difference [16]. Previously, data demonstrated that exercise training improves peak oxygen consumption in heart failure patients by increasing the cardiac output with an unchanged average arteriovenous oxygen difference, suggesting the crucial role of blood flow distribution towards the periphery [17]. In this Special Issue, Miyazaki and colleagues [18] deeply investigated **exertional pathophysiology conditions**, specifically evaluating oxygen extraction measurements following rehabilitation in chronic obstructive pulmonary patients. The same intriguing comparison between pulmonary and cardiac patients’ pathophysiologies was also performed by evaluating different parameters, such as minute ventilation for a given carbon dioxide production, which is known to also have a relevant prognostic power in heart failure patients [19].

## 4. Settings

The home-based model of CR is a feasible proposal for an alternative rehabilitation model, and **Telerehabiliation** is a term used for a set of rehabilitation services provided remotely via telecommunications systems and the Internet which were widely used in the recent era of pandemic restrictions. In a prospective, single-centre, two-arm randomized controlled trial, Batalik and colleagues [20] supported that telehealth CR could be an alternative to usual outpatient CR, demonstrating that the training intensities in a telehealth CR model were similar to those in the usual outpatient CR in low-to-moderate risk coronary artery disease patients.

Moreover, and in more detail, a systemic review and meta-analysis [21] published in this Special Issue provided deep insight into the feasibility of home-based CR with the use of wearable sensors for the telemonitoring and guidance of exercise sessions as an effective “adjunct” or ”alternative” method to improve the accessibility, adherence and participation in CR.

Finally, knowing the importance of risk stratification scores in cardiac patients [22,23], Cabrera-Aguilera and colleagues [24] developed an easy-to-calculate **score** based on cardiopulmonary capacity and left ventricular systolic function with the main goal, of identifying low-risk patients who can safely complete CR in a setting other than the hospital, making CR more accessible to suitable patients.

In conclusion, no matter the reason, whether for measurement via a cardiopulmonary exercise test or for a training session as a part of a cardiac rehabilitation program, it’s time to run!
